# Transarterial chemoembolization with ^125^I seed insertion for multifocal hepatocellular carcinoma

**DOI:** 10.3389/fonc.2024.1384293

**Published:** 2024-04-15

**Authors:** You-Bin Wang, Ying Zhang, Peng-Fei Li, Le Bao, Wen-Tao Zhang

**Affiliations:** ^1^ Department of Interventional Radiology, Xuzhou Cancer Hospital, Xuzhou, China; ^2^ Department of Radiology, Xuzhou Central Hospital, Xuzhou, China; ^3^ Department of Gastrointestinal Surgery, Xuzhou Central Hospital, Xuzhou, China

**Keywords:** hepatocellular carcinoma, multifocal, TACE, ^125^I seed, survival

## Abstract

**Background:**

A common treatment strategy for individuals with multifocal hepatocellular carcinoma (HCC) who are not candidates for surgical resection is transarterial chemoembolization (TACE). Combining TACE with ^125^I seed insertion (ISI) may offer a means of enhancing therapeutic efficacy. The purpose of this study was to compare the therapeutic efficacy of TACE administered with and without ISI for the treatment of multifocal HCC.

**Methods:**

The data from the two centers were analyzed retrospectively. The present study involved 85 consecutive patients with multifocal HCC who underwent TACE between January 2018 and December 2021. Of these patients, 43 were in the combined group, receiving TACE with ISI, and 42 were in the TACE-only group, receiving TACE without ISI. Comparisons of treatment outcomes were made between these groups.

**Results:**

No significant differences in baseline data were observed between these groups of patients. Higher rates of complete (60.5% vs. 33.3%, P = 0.016) and total (93.0% vs. 61.9%, P = 0.001) responses were evident in the combined group compared to the TACE-only group. Median progression-free survival (PFS, 13 vs. 10 months, P = 0.014) and overall survival (OS, 22 vs. 17 months, P = 0.035) were also significantly longer in the combined group than in the TACE-only group. Using a Cox regression analysis, risk variables associated with shorter PFS and OS included Child-Pugh B status (P = 0.027 and 0.004) and only TACE treatment (P = 0.011 and 0.022).

**Conclusion:**

In summary, these findings suggest that, as compared to TACE alone, combining TACE and ISI can enhance HCC patients’ treatment outcomes and survival.

## Introduction

Hepatocellular carcinoma (HCC) accounts for 85–90% of cases of primary liver cancer (1), which is still the third most common cause of cancer-associated death worldwide (2). An estimated 35-40% of HCC patients already have multifocal disease when they are first diagnosed (3–5), with a 5-year overall survival (OS) rate of just 19.5% (3).

Surgical liver resection and liver transplantation are considered the most effective therapy alternatives for patients with multifocal HCC (3). Certain patients are unsuitable for liver resectiondue to the risk of post-operative liver failure caused by the removal of excessive liver tissue, which can result in inadequate hepatic reserves (6, 7). Liver transplantation is also not a standard therapeutic approach owing to limited tissue availability (5).

Another treatment approach, called transarterial chemoembolization (TACE), is frequently used in individuals with multifocal HCC who have incurable disease (6, 7). Treatment with TACE alone, however, generally results in poor multifocal HCC patient outcomes including a 3-year OS rate of just 18.1-26.7% (3, 6, 7). Efforts to prolong OS and progression-free survival (PFS) following TACE often entail further percutaneous ablation to disrupt target tumors (8). Tumors near the diaphragm, major arteries, liver surface, or gallbladder should not be treated with percutaneous ablation (9). Furthermore, patients with multifocal HCC might not be able to handle several rounds of percutaneous ablation. Antiangiogenetic drug, such as sorafenib, has been used after TACE to improve the clinical effectiveness of TACE (10, 11), and the 1-year OS rate could be improved with 8% by using the sorafenib (11). The sorafenib may decrease TACE-induced vascular endothelial growth factor overproduction, thus complementing TACE fordisease control (11). However, the cost of sorafenib is high, and therefore, not all families can afford it.

Currently, ^125^I seed insertion (ISI) is often employed to treat HCC cases that are not good candidates for ablation (5, 9). Relative to ablation procedures, ISI has several advantages, including better patient tolerance and its ability to deliver localized therapeutic effects (5, 9). According to a recent meta-analysis, combined TACE and ISI could improve the total treatment response rate from 51% to 80%, with the 3-year OS rate from 14.6% to 34.9% when compared to TACE alone (9). However, the number of tumors each patient had was not considered when stratifying the patients in the earlier trials. As a result, we cannot obtain specific results from patients with multifocal HCC. The advantages of combining TACE and ISI in patients with multifocal HCC have not received much attention despite the necessity for such a study.

In order to manage multifocal HCC, the current study assessed the therapeutic efficacy of TACE when used alone or in combination with ISI.

## Materials and methods

### Study design

This study retrospectively examined patients from two centers, namely Xuzhou Cancer Hospital and Xuzhou Central Hospital. The study received approval from the Institutional Review Boards of both hospitals, which exempted the need for informed consent.

This study included 85 consecutive multifocal HCC patients treated with TACE alone (n = 42) or combined with ISI (n = 43) between January 2018 and December 2021.

**Figure 1:**
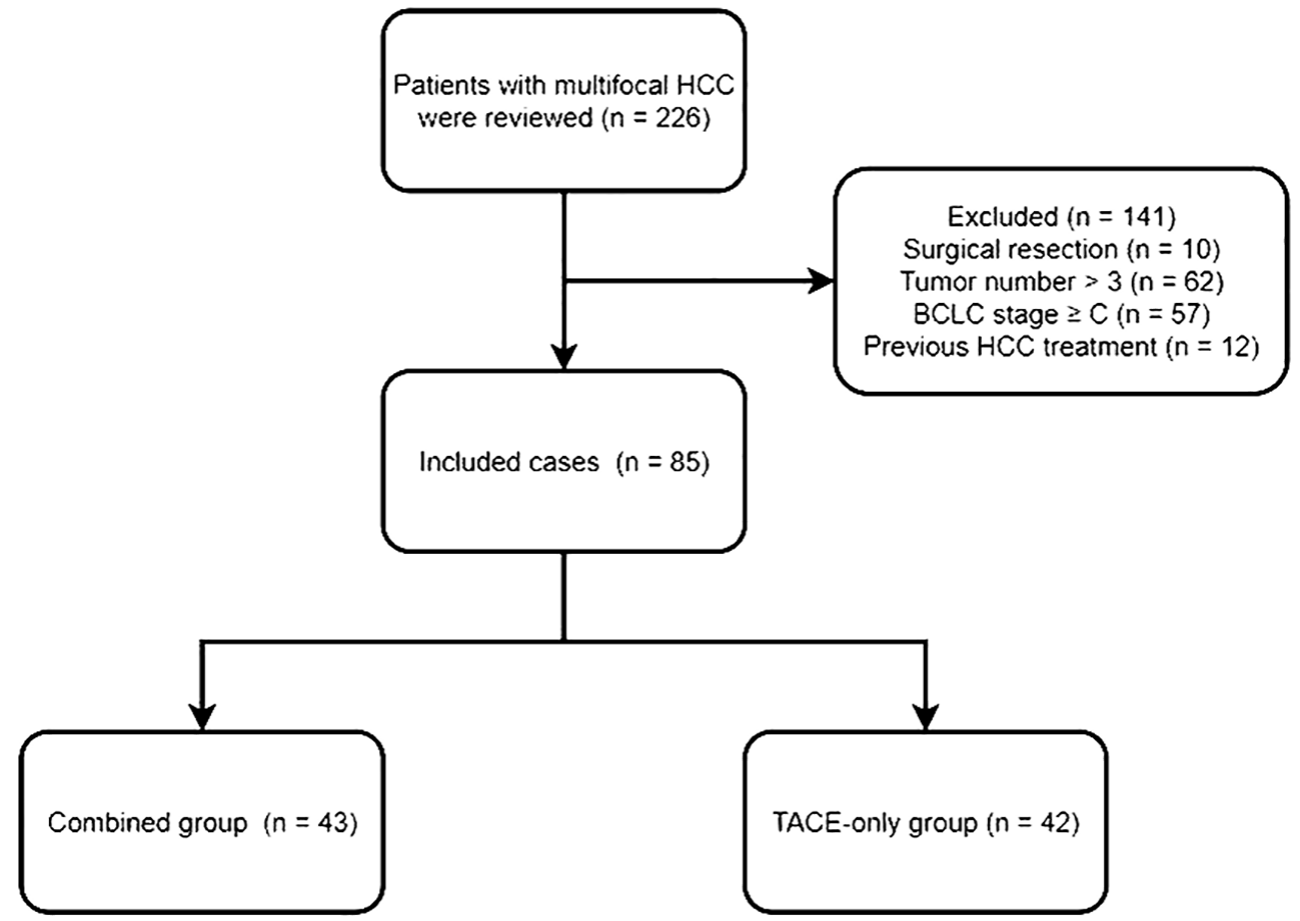
The flowchart of this study.

Eligible patients had to (a) be diagnosed with multifocal HCC, (b) be unable or unwilling to undergo surgical treatment, and (c) have 3 or fewer tumors. Patients with (a) a history of prior HCC treatments, (b) a BCLC stage of C or greater, or (c) a life expectancy < 3 months were excluded. The flowchart of this study is shown in **Figure 1**.

Age, gender, hepatitis types, alpha-fetoprotein (AFP) level, liver function tests, stages of Barcelona Clinic Liver Cancer (BCLC), and results from preoperative computed tomography (CT) and/or magnetic resonance image (MRI) scans were among the data that were collected.

### Diagnosis

Patients were diagnosed with HCC as per the AASLD guidelines (12). Typical MRI or CT manifestations of HCC consisted of an enhancement pattern associated with arterial hypervascularity and venous/delayed phase washout (12). In cases when typical imaging findings were lacking, a percutaneous biopsy was performed. The number and diameter of the tumors were assessed using preoperative MRI or CT images. All images were independently analyzed by 2 radiologists with 6 and 12 years of experience in abdominal diagnosis. In case of a disagreement, a third radiologist with 16 years of experience was consulted to make the final decision.

### TACE treatment

TACE was performed under the fluoroscopic guidance with local anesthesia. In summary, angiography confirmed the tumor site after a 5F RH catheter (Angiopointer, Xiangxiang, China) was inserted into the celiac artery. A 2.7F microcatheter (Terumo, Fujinomiya, Japan) was then introduced into the blood-supplying artery for each tumor, and a mix of 5-fluorouracil (150 mg), mitomycin (10 mg), epirubicin (50 mg), and lipiodol (10–20 mL) was introduced for TACE. A one-stage TAEC procedure was used to embolize all tumors. The microcatheter was then removed, and angiography was repeated with the 5F RH catheter to confirm TACE efficacy.

### ISI treatment

A treatment-planning system (TPS; Fei-Tian, Beijing, China) was loaded with abdominal CT images, and it was then used to determine the optimal number and distribution of ^125^I seeds (model-6711, length: 4.5 mm, diameter: 0.8 mm, half-life: 59.6 days, activity: 0.6–0.8 mCi). ISI was typically conducted within 14 days following TACE and was performed with a 64-row CT instrument (Siemens, Erlangen, Germany) for guidance. Based on preoperative TPS reports, patient positioning, puncture sites, and needle pathways were established. Multiple 18G puncture needles were introduced into target tumors, following the insertion of the ^125^I seeds spaced 5-10 cm apart as per the established treatment strategy. The seeds were then kept inside the needle routes by removing the needles. All target tumors underwent seed insertion using a single-stage ISI technique.

### Follow-up

Patients underwent follow-up after 1, 3, and 6 months post-treatment and every 6 months after that until death or study end (November 30, 2023). If a patient was lost in follow-up, the follow-up ended at the date of the last visit. Follow-up consisted of routine blood testing, analyses of liver function, contrast-enhanced CT/MRI, and AFP testing.

### Assessments

The mRECIST criteria were used to evaluate patient treatment responses at 1 month post-treatment. Complete response (CR) was defined by the absence of any detectable enhancement in target lesions, while partial response (PR) was defined by a ≥ 30% reduction in the sum of the diameters of visible target lesions relative to baseline. Progressive disease (PD) was defined by a ≥ 20% increase in the sum of the diameters of visible target lesions relative to baseline, whereas all other cases were classified as stable disease (SD) (13). Total response rates were calculated by adding together CR and PR cases. When enhancement was evident within treated tumors, TACE procedures were repeated. PFS was measured from treatment until disease progression or death, and OS was measured from treatment until death or the last follow-up. Treatment-related toxicities were assessed with the Common Terminology Criteria for Adverse Events version 4.0 (CTCAEv4) (14).

### Statistical analyses

SPSS 16.0 (SPSS, Inc., IL, USA) was employed to analyze all data. Quantitative data were reported as means ± standard deviation if normally distributed and median (Q1; Q3) if skewed, with respective comparisons via t-tests and Mann-Whitney U tests. The categorical data were analyzed using either χ^2^ or Fisher’s exact tests. OS and PFS were compared via Kaplan-Meier curves and log-rank tests. Factors related to OS and PFS were identified via multivariate Cox regression analyses. The factors with P < 0.1 on univariate Cox regression analysis were included into the multivariate Cox regression analysis. P < 0.05 was used to define significance.

## Results

### Patient characteristics

**Figure 2:**
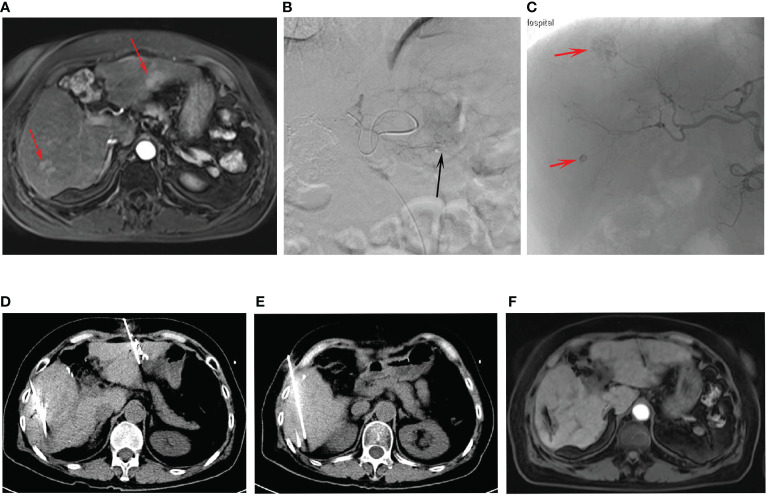
The procedures of combined treatment for multifocal HCC. **(A)** The preoperative contrast-enhanced MRI showed the tumors (arrows) at the left and right lobes. **(B)** The TACE for the left lobe tumor (arrow). **(C)** The TACE for the right lobe tumors (arrows). **(D, E)** The procedures of CT-guided ISI after TACE for the multifocal HCC. **(F)** The post-operative contrast-enhanced MRI indicated the treatment response was CR.

This study enrolled 85 patients with multifocal HCC, of whom 43 were treated with TACE and ISI in combination (**Figure 2**), while 42 were treated with TACE only. Among the 85 patients, 78 patients (91.8%) exhibited typical imaging manifestations of HCC and 7 (8.2%) patients required percutaneous biopsy due to non-typical imaging manifestations. The combined group included 33 and 10 patients with 2 and 3 tumors, respectively, while 30 and 12 patients in the TACE-only group exhibited 2 and 3 tumors. None of the patients encountered significant complications related to the ISI or TACE procedures. **Table 1** displays the initial data of the patients. These two groups had no significant difference in the baseline patient data.

**Table 1:** Baseline data of the included patients.

	Combined treatment (n = 43)	TACE only (n = 42)	P value
Age (y)	58.5 ± 6.7	60.8 ± 10.1	0.089
Gender			0.071
Male	39	32	
Female	4	10	
Hepatitis types			0.079
B	26	32	
C	8	8	
None	9	2	
AFP level before treatment (ng/ml)	169.8 (Q1: 18.2, Q3: 283.0)	145.7 (Q1: 8.5, Q3: 640.0)	0.916
AFP level after treatment (ng/ml)	61.5 (Q1: 10.2, Q3: 254.9)	32.3 (Q1: 4.6, Q3: 361.0)	0.521
Child-Pugh class			0.955
A	34	33	
B	9	9	
BCLC stage			0.955
A	9	9	
B	34	33	
Number of tumors			0.576
2	33	30	
3	10	12	
Tumor diameter (cm)	3.1 (Q1: 2.3, Q3: 4.6)	2.9 (Q1: 1.8, Q3: 3.8)	0.083
Target or immune therapy	4	6	0.707

AFP, alpha-fetoprotein; BCLC, Barcelona Clinic Liver Cancer; TACE, transarterial chemoembolization.

### Treatment response

The combined group exhibited significantly higher CR (60.5% vs. 33.3%, P = 0.016) and total response (93.0% vs. 61.9%, P = 0.001) rates relative to the TACE-only group (**Table 2**). In comparison, comparable rates of PD were evident in both groups (6.9% vs. 0%, P = 0.241).

**Table 2:** Treatment response between 2 groups.

	Combined treatment (n = 43)	TACE only (n = 42)	P value
CR	26 (60.5%)	14 (33.3%)	0.016
PR	14 (32.6%)	12 (28.6%)	0.690
SD	0 (0%)	16 (38.1%)	0.001
PD	3 (6.9%)	0 (0%)	0.241
Total response (CR + PR)	40 (93.0%)	26 (61.9%)	0.001

CR, complete response; PD, progressive disease; PR, partial response; SD, stable disease.

### Treatment-related toxicity

The most common forms of treatment-associated toxicity in the present study included fever (37.2% vs. 36.6%, P = 0.953), vomiting (34.9% vs. 39.0%, P = 0.694), and myelosuppression (23.2% vs. 19.5%, P = 0.676), all of which occurred with similar frequency in both groups (**Table 3**). There were no cases of CTCAEv4 grade 3 or 4 toxicity observed in any of these patients.

**Table 3:** Toxicity effects between 2 groups.

	Combined treatment (n = 43)	TACE only (n = 42)	P value
Fever	16 (37.2%)	15 (36.6%)	0.953
Vomit	15 (34.9%)	16 (39.0%)	0.694
Myelosuppression	10 (23.2%)	8 (19.5%)	0.676

### Patient survival

**Figure 3:**
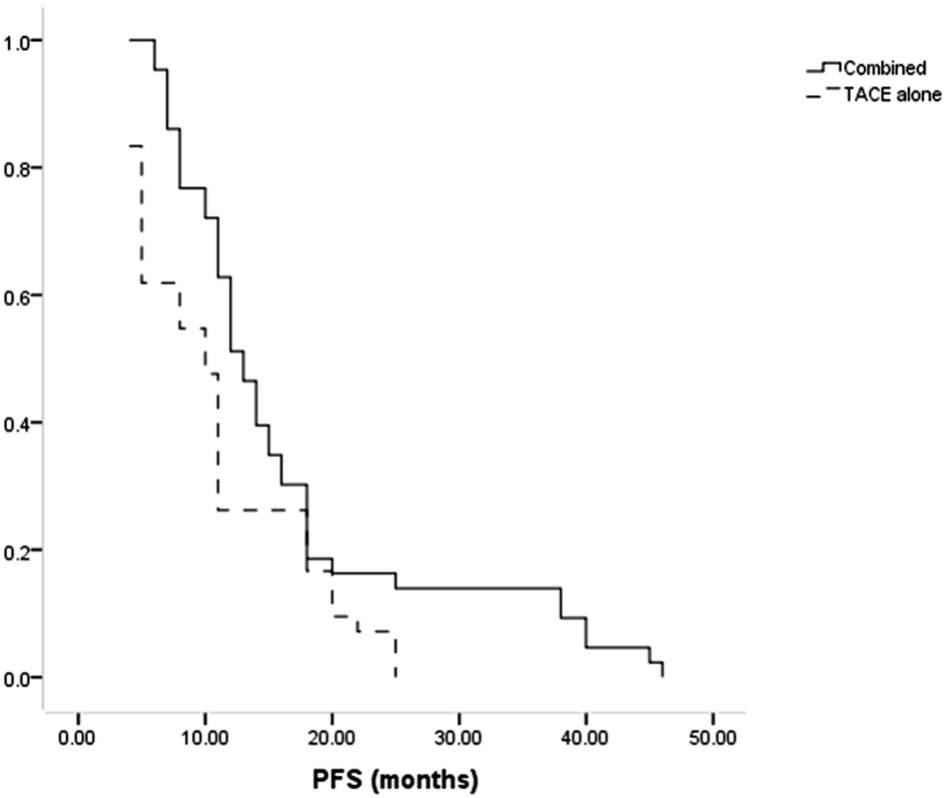
The comparison of PFS between combined and TACE alone groups.

Patients in the combined group had a higher median PFS than in the TACE-only group (13 months vs. 10 months, P = 0.014, **Figure 3**). The combination treatment group showed cumulative 1- and 3-year PFS rates of 62.8% and 14.0%, respectively, compared to equivalent rates of 26.2% and 0.0% in the TACE-only group. Cox regression analyses led to the identification of Child-Pugh B status (P = 0.027) and treatment with TACE alone (P = 0.011) as risk factors for shorter PFS (**Table 4**).

**Table 4:** Predictors of progression-free survival.

Variables	Univariate analysis			Multivariate analysis		
	Hazard ratio	95% CI	P value	Hazard ratio	95% CI	P value
Age (y)	0.997	0.970-1.025	0.827			
Gender						
Male	1					
Female	0.644	0.368-1.128	0.124			
Hepatitis types						
None	1					
B	1.485	0.753-2.928	0.254			
C	0.945	0.431-2.074	0.889			
AFP level before treatment	1.000	1.000-1.000	0.286			
AFP level after treatment	1.000	1.000-1.000	0.302			
Child-Pugh class						
A	1			1		
B	1.641	0.951-2.830	0.075	1.887	1.074-3.315	0.027
BCLC stage						
A	1					
B	0.648	0.378-1.110	0.114			
Number of tumors						
2	1					
3	1.009	0.619-1.645	0.971			
Largest tumor diameter	1.083	0.924-1.270	0.325			
Treatment options						
Combined treatment	1			1		
TACE only	1.660	1.064-2.591	0.026	1.807	1.146-2.850	0.011

AFP, alpha-fetoprotein; BCLC, Barcelona Clinic Liver Cancer; TACE, transarterial chemoembolization.

**Figure 4:**
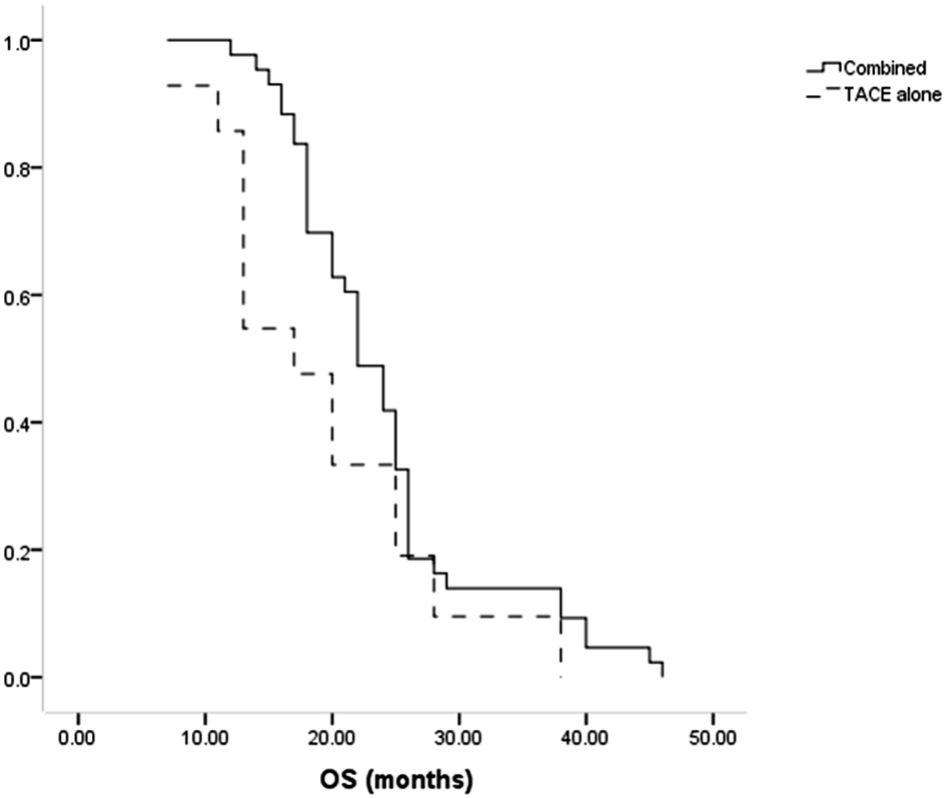
The comparison of OS between combined and TACE alone groups.

All patients died during the follow-up. The median OS of patients in the combination group was longer compared to the TACE-only group (22 months vs. 17 months, P = 0.035, **Figure 4**). Cumulative rates of 1- and 3-year OS in the combined group were 97.7% and 14.0%, as compared to 85.7% and 9.5% in the TACE-only group. Univariate and multivariate analyses identified Child-Pugh B status (P = 0.004) and treatment with TACE alone (P = 0.022) as risk factors related to shorter OS (**Table 5**).

**Table 5:** Predictors of overall survival.

Variables	Univariate analysis			Multivariate analysis		
	Hazard ratio	95% CI	P value	Hazard ratio	95% CI	P value
Age (y)	0.994	0.965-1.023	0.671			
Gender						
Male	1					
Female	1.030	0.572-1.855	0.921			
Hepatitis types						
None	1					
B	1.670	0.858-3.250	0.131			
C	0.920	0.420-2.017	0.835			
AFP level before treatment	1.000	1.000-1.000	0.115			
AFP level after treatment	1.000	1.000-1.000	0.108			
Child-Pugh class						
A	1			1		
B	2.064	1.184-3.597	0.011	2.345	1.320-4.164	0.004
BCLC stage						
A	1					
B	0.726	0.426-1.237	0.726			
Number of tumors						
2	1					
3	0.936	0.574-1.526	0.790			
Largest tumor diameter	1.047	0.904-1.212	0.539			
Treatment options						
Combined treatment	1			1		
TACE only	1.530	0.987-2.370	0.057	1.692	1.081-2.649	0.022

AFP, alpha-fetoprotein; BCLC, Barcelona Clinic Liver Cancer; TACE, transarterial chemoembolization.

## Discussion

The range of disease morphologies associated with multifocal HCC, from small oligo nodular tumors to widespread disease, makes treatment challenging (3). Many recent reports have suggested that surgical resection can afford multifocal HCC patients better survival than TACE (3, 6, 7). Nevertheless, patients with multifocal HCC must meet certain requirements to be considered for surgery. These include having tumors limited to a single lobe and having normal liver function (15). TACE continues to be the first-line treatment for patients with multifocal HCC that is incurable (16).

ISI and other brachytherapy strategies including transarterial radioembolization and selective internal radiation therapy have been employed to treat HCC (10, 16, 17). TACE is possible with ISI since it is carried out via a CT-guided percutaneous route. The advantage of ISI over conventional radiotherapeutic intervention is that it may be easily regulated while providing modest doses of radiation directly to the interior sections of the tumor for an extended length of time (18).

The present study examined patients with multifocal HCC treated with TACE alone versus ISI for clinical results. Compared to the TACE_only group, the combined group showed higher CR and total response rates, with a total response rate of up to 93.0%. These results suggested that ISI can further aid in destroying tumor tissue beyond the efficacy afforded by TACE. When attempting to treat HCC tumors with a poor blood supply, TACE generally cannot achieve embolization completely, and ISI may be an effective supplemental treatment strategy in this context (9, 11). Furthermore, the CR (60.5%) and total response (93.0%) rates in combined group in this study were comparable to those (59.0% and 92.3%) in a previous study regarding the combined TACE and ISI for HCC (5). These findings may indicate that the number of tumors did not influence the treatment response of combined TACE and ISI.

Patients with TACE are frequently treated with percutaneous ablation techniques, and ablation is usually advised for those with HCC lesions ≤ 3 cm (19). Patients with multifocal HCC might not be able to handle one-stage multi-ablation treatments because of the severe discomfort they can induce (9). ISI is associated with only minor levels of pain, providing a further advantage over ablative therapies (9), and this low level of pain is particularly beneficial for multifocal HCC patients.

The potential of combined treatment to manage the disease over the long term was demonstrated by the longer OS and PFS intervals compared to with TACE alone. ^125^I seeds can eliminate tumor tissues while enhancing tumor chemosensitivity to improve tumor control in the long term (20). Cox regression analysis revealed that using TACE alone was linked to decreased OS and PFS. Child-Pugh B status was also linked to a higher likelihood of shorter PFS and OS, which aligns with the understanding that Child-Pugh B status signifies impaired liver function.

IThe median overall survival of patients in the combined treatment group was 22 months, lower than the previously reported median OS of 31-41 months for multifocal HCC patients who underwent surgical resection (3, 7). Similarly, the 3-year OS rate in this study (14.0%) is also lower than the 51.5-64.3% reported previously for surgically treated multifocal HCC (2, 3). The results do not offer conclusive evidence on whether surgical resection is superior to the combination of TACE and ISI. This is because patients eligible for surgical resection have lower-stage tumors and better liver function compared to those who cannot undergo surgery.

Similar rates of treatment-related toxicity were observed in both groups, indicating that ISI did not contribute to any increased levels of toxicity beyond what is already inherent to the TACE technique. This is probably because the selected ^125^I seed distribution was deliberately planned before the operation utilizing the TPS.

This study is subject to certain limitations. For one, as a retrospective study these results are susceptible to a high potential for bias, emphasizing a need for additional prospective randomized controlled trials. Although these patients were obtained from two centers, variations in the experience of interventional radiologists at these institutions could have introduced some possibility for further bias. Finally, the sample size was partially small. While no relationships between patient survival and hepatitis types, AFP level, Child-Pugh class, BCLC stage, number of tumors, or tumor diameter were detected, the additional large-scale analysis will be vital to validate these findings.

## Conclusions

In conclusion, these findings indicate that using combination of TACE and ISI can enhance therapeutic response and survival rates in patients with multifocal HCC compared to using TACE alone.

## Data availability statement

The raw data supporting the conclusions of this article will be made available by the authors, without undue reservation.

## Ethics statement

The studies involving humans were approved by Xuzhou Cancer Hospital, Xuzhou Central Hospital. The studies were conducted in accordance with the local legislation and institutional requirements. The ethics committee/institutional review board waived the requirement of written informed consent for participation from the participants or the participants’ legal guardians/next of kin because this is a retrospective study.

## Author contributions

Y-BW: Funding acquisition, Methodology, Writing – original draft. YZ: Data curation, Formal analysis, Writing – review & editing. P-FL: Formal analysis, Writing – original draft. LB: Formal analysis, Writing – review & editing. W-TZ: Supervision, Validation, Writing – original draft, Writing – review & editing.
